# Advancing basic and preclinical spine research: Highlights from the ORS PSRS 6th International Spine Research Symposium

**DOI:** 10.1002/jsp2.1308

**Published:** 2023-11-21

**Authors:** Lachlan J. Smith, John T. Martin, Makarand V. Risbud

**Affiliations:** ^1^ Department of Orthopaedic Surgery, Perelman School of Medicine University of Pennsylvania Philadelphia Pennsylvania USA; ^2^ Translational Musculoskeletal Research Center, Corporal Michael J. Crescenz VA Medical Center Philadelphia Pennsylvania USA; ^3^ Department of Orthopedic Surgery Rush University Medical Center Chicago Illinois USA; ^4^ Department of Orthopaedic Surgery Thomas Jefferson University Philadelphia Pennsylvania USA

## Abstract

The sixth biennial ORS PSRS International Spine Research Symposium was held from November 6 to 10, 2022, at Skytop Lodge in northeastern Pennsylvania, USA. Organized jointly by the Orthopaedic Research Society and the Philadelphia Spine Research Society, the symposium attracted more than 200 participants from 15 different countries who came together to share the latest advances in basic and preclinical spine research. Following the symposium, selected participants were invited to submit full‐length manuscripts to this special issue of *JOR Spine*.

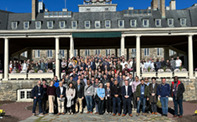

## 1 INTRODUCTION

**FIGURE 1:**
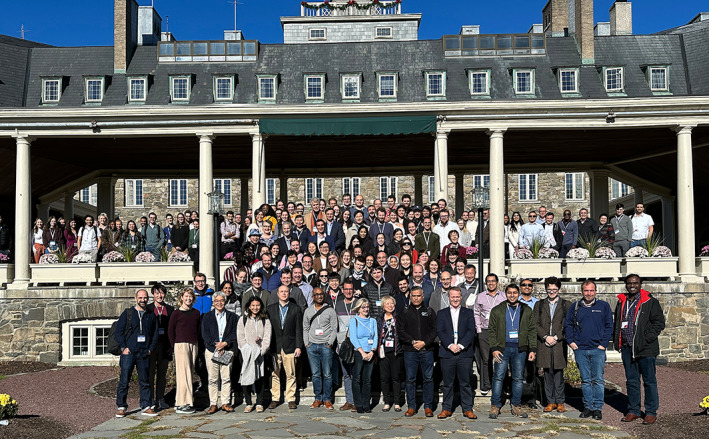
ORS PSRS 6th International Spine Research Symposium attendee group photo.

The sixth biennial ORS PSRS International Spine Research Symposium was held from November 6 to 10, 2022, at Skytop Lodge in northeastern Pennsylvania, USA (https://www.ors.org/psrs-2022/). Organized jointly by the Orthopaedic Research Society (ORS) and the Philadelphia Spine Research Society (PSRS), the symposium attracted more than 200 participants from 15 different countries who came together to share the latest advances in basic and preclinical spine research (Figure 1). Spanning three‐and‐a‐half days, the program included 20 invited faculty presentations, and 29 podium and 124 poster presentations predominantly from trainees. Session topics included: Development and Homeostasis, Pain, Pathobiology, Crosstalk, Imaging, Biomechanics, and Next Generation Treatments. The 5th PSRS Lifetime Achievement Award was presented to Dr Judith Hoyland, for her pioneering work on the biology and pathophysiology of the intervertebral disc. The most exceptional trainee presentations were recognized by 4 podium and 12 poster awards sponsored by the PSRS. The ORS Spine Section sponsored awards recognizing the two most innovative presentations and the two most outstanding early‐stage investigators. Additionally, seven diversity fellowships were awarded to support attendance by trainees from underrepresented minorities. Members of the Back Pain Consortium (BACPAC) led an engaging workshop on the topic of knowledge organizing technologies to advance transdisciplinary back pain research. Between scientific sessions, participants networked and socialized in the beautiful late fall surroundings of Pennsylvania's Pocono Mountains. A highlight was an evening campfire with smores, a particular novelty for attendees from outside North America. As is tradition, the symposium concluded with an entertaining, spine‐themed quiz night organized and moderated by trainee members of the ORS Spine Section. In this Special Issue of *JOR Spine*, we present exceptional original research and review articles submitted by Symposium award winners, discussion leaders and invited faculty speakers. A unifying theme across each of these articles is the importance of recognizing crosstalk across tissues, organs, and disciplines in order to better understand and treat disc degeneration and back pain.

## 2 SPECIAL ISSUE HIGHLIGHTS

Biopsychosocial modeling has emerged as a potentially powerful tool for holistic diagnosis and prognosis of low back pain, and for tailoring patient‐specific treatments. In their article, Lotz et al. explore the current capabilities of knowledge integration technologies to synthesize biomedical literature and depict multimodal relationships reflected in these biopsychosocial models, and highlight limitations, implementation details, and future areas of research to improve performance.^1^

Cross disciplinary approaches in basic and translational research, for example, those that examine the interplay between biology and biomechanics, are critical to advancing the understanding of disc degeneration pathophysiology. Using a rabbit model, Fainor et al. show that perturbations in disc biomechanics not only cause local pathological remodeling in structures such as the vertebral endplates, but also in adjacent structures such as the facet joints.^2^ Easson et al. identified the ion channel transient receptor potential vanilloid 4 (TRPV4) as performing a critical role in mediating the interplay between inflammation and loading in mouse intervertebral discs.^3^

The cartilaginous endplates perhaps more than any other disc substructure illustrate the central role of biomechanical and biological interplay in disc function, both facilitating pressurization by restricting fluid flow and regulating the passage of nutrients to cells within the avascular nucleus pulposus. In a comprehensive review, Crump et al. outline the critical role of the cartilaginous endplates in disc health and degeneration.^4^

There is increasing recognition of the important role of sex‐dependent differences in anatomy and physiology in disc health. Using a rat model, Kenawy et al. demonstrate that the biomechanical, inflammatory, biochemical, and histological injury responses of the disc are sex‐dependent,^5^ while Hutchinson et al. show that sex differences impact age‐related degenerative changes to the disc in mice.^6^

Biomarkers are not only invaluable diagnostic and prognostic tools for disc degeneration and low back pain, but also provide mechanistic insights into the interplay between local tissue changes and systemic changes such as those of the central nervous and immune systems. Pereira et al. review current imaging and molecular biomarker candidates for back pain, and their utility in improving patient care.^7^

Finally, recognizing the importance of tissue crosstalk is also critical in the development of next generation treatments for disc degeneration and back pain. Using an ovine model, Panebianco et al. demonstrate how different biomaterial‐based approaches for disc repair differentially impact component substrates such as the nucleus pulposus and vertebral endplates.^8^ Koga et al. use finite element modeling to demonstrate the important role of vertebral body geometry in the failure of disc implants, independent of implant strength.^9^

## CONFLICT OF INTEREST STATEMENT

Lachlan J. Smith, John T. Martin, and Makarand V. Risbud are members of the JOR Spine Advisory Review Board. Lachlan J. Smith is a current ORS Spine Section officer.
